# Divergent Roles of *SOG* Family Genes in Salt Tolerance: A Comparative Genomics Study Between Barley and Rice

**DOI:** 10.3390/plants15111620

**Published:** 2026-05-25

**Authors:** Yuxi Weng, Xintong Zheng, Xiaohan Xu, Zhengxing Zhou, Kerun Chen, Hongkai Wu, Liangbo Fu

**Affiliations:** College of Advanced Agricultural Sciences, Zhejiang A&F University, Hangzhou 311300, China

**Keywords:** barley, rice, *SOG*, salt tolerance, comparative genomics

## Abstract

Salt stress is one of the major abiotic stresses limiting the yield of agriculture production worldwide. Rice is an aquatic summer crop, while barley represents a drought winter crop. Both are classified as diploid sequenced crops within the Poaceae family, and are vital staples in the world. As a plant-specific transcriptional regulator, suppressor of gamma response (*SOG*) plays crucial roles in plant adaptation under abiotic stresses by repairing DNA damage pathway. However, little research has reported the function of *SOGs* in barley and rice. This study presents the first genome-wide identification and comparative analysis of the *SOG* gene family in barley and rice, two cereal crops with contrasting salt tolerance. A total of 97 *HvSOGs* and 74 *OsSOGs* were identified in the genome of barley and rice, which were divided into three subfamilies. There was significant variation between barley and rice in terms of gene structures, motif compositions, gene duplication, and cis-elements. Notably, rice may have suffered stronger purifying selection pressure than barley, whereas the proportion of *SOGs* with stress-related cis-elements was significantly higher in barley than in rice. The expression patterns of *SOGs* in barley and rice tissues under salt stress indicated that barley’s stronger salt tolerance was largely due to an energy-saving strategy in shoots. Moreover, homologous gene similarity comparison with sea barleygrass suggested that gene loss and possible functional divergence during evolution may contribute to salt sensitivity in rice. Functional validation of a differentially expressed *OsSOG17* gene confirmed its positive regulatory role in salt tolerance. Our findings uncover an energy-saving strategy as a potential mechanism underlying differential salt tolerance, and functionally link a *SOG* gene to salt stress responses in rice.

## 1. Introduction

Soil salinity is one of the major abiotic stresses, resulting in a considerable reduction in crop production on a global scale [1,2]. According to statistics, the total area of salt-affected soils spans approximately 10.0 × 10^8^ hm^2^, encompassing 20% of arable lands and nearly half of irrigated lands [3]. China, specifically, harbors over 1.0 × 10^8^ hm^2^ of saline soils, surpassing the extent found in other countries and regions [4]. Unfortunately, the saline soils exhibit high salt concentrations and are characterized by the difficulty of comprehensive management. Moreover, due to the worsening impacts of climate change and inadequate irrigation practices, the issue is trending towards expansion [5], posing a significant threat to the sustainable development of agriculture. Therefore, elucidating the mechanisms of salt tolerance in crops and breeding salt-tolerant crop varieties is essential to unlock the productivity potential of saline land and ensure the significant demand for the sustainable development of agriculture and food security.

On the other hand, both barley and rice, classified as diploid crops within the Poaceae family, are vital staples worldwide, undergoing widespread cultivation and holding considerable economic significance. However, rice displays poor salt tolerance, with its threshold value for tolerant concentration being less than 150 mM [6]. Compared to rice, barley has a short growth period, wide adaptability, and strong salt tolerance, capable of surviving under 300 mM salt concentration [7,8]. In particular, the high-quality genome sequence of barley has been decoded [9,10], making it an ideal model crop for research on genetic breeding of crops with strong stress tolerance. Clearly, barley’s exceptional salt tolerance offers valuable insights for enhancing salt tolerance in rice. Thus, it is imperative to identify and compare the salt-tolerant genes of gene families between barley and rice.

Transcriptional regulation is a crucial regulatory mechanism commonly found in the formation of plant stress tolerance, and transcription factors are key factors in this process [11]. Presently, multiple transcription factor families with stress-tolerance functions have been identified in plants, such as NAC, bHLH, bZIP, MYB and WRKY [12,13,14]. They modulate the expression of downstream target genes by binding to cis-elements in the promoter region either positively or negatively, enabling plants to modulate their responses to both internal signals and external environmental stimuli [15]. For example, Li et al. revealed that MYB transcription factors have the ability to positively regulate anthocyanin biosynthesis, thereby influencing plant resistance to diverse stresses [16]. Additionally, WRKY transcription factors have been identified to play critical roles in abiotic stress responses [17,18]. Typically, ZmWRKY17 has been found to negatively regulate salt tolerance through the ABA signaling pathway [19]. Furthermore, research indicates that PeTCP10 has the capability to enhance salt tolerance in transgenic *arabidopsis* during the vegetative growth stage, but it has been observed to increase salt sensitivity during both germination and seedling stages [20]. Clearly, identifying and elucidating the functions of transcription factors in abiotic stress is of great significance for breeding stress-tolerant crop varieties.

Among these stress-related transcription factors, SOG (suppressor of gamma response) transcription factors are plant-specific proteins that contain a highly conserved NAM (no apical meristem) domain. This domain is characterized by a unique tertiary structure that facilitates sequence-specific DNA binding and protein–protein interactions. The NAM domain plays a vital role in the cell-cycle response to gamma radiation, primarily by recognizing and binding to specific promoter elements of downstream target genes involved in the DNA damage repair pathway [21]. Upon activation, SOGs function as master regulators of the DDR (DNA damage response) network. They have the capability to transcriptionally regulate hundreds of genes during DNA damage, orchestrating a coordinated cellular program that includes cell-cycle checkpoint activation, DNA repair, and, if damage is irreparable, programmed cell death [22]. This hierarchical regulatory role positions *SOGs* at the core of the plant’s genomic integrity surveillance system. On the other hand, salt stress is known to cause secondary oxidative stress in plants, leading to the accumulation of reactive oxygen species (ROS) such as hydrogen peroxide and superoxide anions. Elevated ROS levels can directly damage DNA by causing strand breaks, base modifications, and crosslinks, thereby threatening genome integrity. The DDR network is therefore critical for plant survival under saline conditions. SOG transcription factors orchestrate the expression of hundreds of genes involved in cell-cycle arrest, DNA repair, and, if necessary, programmed cell death. In *arabidopsis*, SOG1 has been shown to be rapidly activated by genotoxic stress and to play a central role in maintaining genomic stability. Given that salt stress induces DNA damage through oxidative pathways, it is plausible that *SOGs* may also contribute to salt tolerance by coordinating DNA repair and stress adaptation mechanisms. However, whether and how SOGs participate in salt stress responses in cereals remains largely unknown.

Additionally, *SOG* homologous genes are highly conserved across *spermatophyta* (seed plants), indicating that their functions in DNA damage control have been preserved throughout land plant evolution [23]. For example, orthologs of *SOG1* have been identified in diverse species ranging from *arabidopsis* to rice, barley, and even gymnosperms, suggesting that the DDR regulatory network mediated by SOG proteins is an ancient and essential mechanism for plant survival under genotoxic stress conditions. Despite this high conservation, only a few SOG transcription factors have been functionally characterized to date, and these exhibit a variety of roles beyond the canonical DDR response. For instance, Nishizawa et al. demonstrated that SOG1 (AT1G25580) in *arabidopsis* not only regulates the expression of DDR-related genes but also directly influences higher-order chromatin organization. Specifically, SOG1 can modulate chromosome structure and mobility in plants, thereby facilitating the accessibility of DNA repair machinery to damaged sites [24]. This regulatory function enables plants to effectively cope with DNA damage, ensure cell survival, and uphold genome stability, processes that are particularly critical in meristematic tissues where active cell division occurs.

Importantly, SOG1 also plays a role in regulating the cell cycle of root tips. In *arabidopsis*, loss-of-function *sog1* mutants exhibit altered root meristem activity, leading to changes in the differentiation and elongation rates of root cells. This finding suggests that SOG1 integrates DNA damage signals with developmental programs, thereby modulating root growth in response to environmental stresses that cause DNA lesions. Furthermore, emerging evidence indicates that SOG1 may interact with the transcription factor SnRK1 (sucrose non-fermenting-related kinase 1), a central energy sensor in plants. This interaction potentially coordinates plant adaptation and growth development under low-energy environments [25]. For example, under energy depletion conditions (such as prolonged darkness or carbon starvation), SnRK1 becomes activated and can directly associate with SOG1 to fine-tune the expression of stress-responsive genes, thereby adjusting metabolic activity and cell-cycle progression to conserve energy. This crosstalk between DNA damage signaling and energy homeostasis highlights the multifaceted nature of SOG proteins, which not only safeguard genomic integrity but also help optimize resource allocation under adverse conditions. Additionally, in our present study [26], RNA-seq analysis of barley and rice under salt stress revealed that many *HvSOGs* and *OsSOGs* are significantly up-regulated or down-regulated. Some homologous *SOGs* exhibit opposite expression patterns in barley and rice. Obviously, SOG transcription factors effectively regulate plants under adverse conditions, thus playing a pivotal role in plant growth and development.

As an important food crop, rice genomes (such as cv. Nipponbare, japonica and cv. 9311, indica) have been fully sequenced and assembled [27,28,29,30]. On the other hand, the high-quality genomes of barley (cv. Morex, reference genome variety; cv. Golden Promise, transformation variety) have also been successfully deciphered. However, the *SOG* gene family in either barley or rice has not been identified yet, and its roles in salt tolerance remain unclear. In this study, 97 and 74 *SOGs* were identified in the barley and rice genome, respectively. In addition, we systematically analyzed their phylogenetic relationships, motif conservation, gene structures, chromosome distributions and cis-elements. Moreover, we analyzed the expression profiles of *SOGs* in different tissues of barley and rice under salt stress, and explored their evolutionary origins in comparison with highly salt-tolerant plant species. Meanwhile, we validated the function of one of the *SOGs* in rice. These findings will offer useful information for further research on molecular mechanisms of *SOGs* in salt tolerance and molecular breeding in both barley and rice.

## 2. Results

### 2.1. Identification and Phylogenetic Analysis of SOGs in Barley and Rice

In the genomes of barley and rice, a total of 97 and 74 proteins with a specific NAM domain (Pfam02365) were identified as the members of the *SOG* family according to *arabidopsis SOGs* (Tables S1–S3). The first amino acid sequence of each protein was selected for subsequent analysis. Detailed information of gene names, IDs, amino acid lengths, molecular weights (MW), and isoelectric points (pI) is listed in Tables S2 and S3. In barley, the amino acid lengths of 97 SOGs ranged from 171 to 2425, with molecular weights varying from 19.2 to 88.0 kDa. As for rice, the lengths of 74 SOGs ranged from 103 to 2190, and molecular weights varied from 11.5 to 80.1 kDa. The coding sequences of each gene are also listed in Tables S2 and S3.

**Figure 1:**
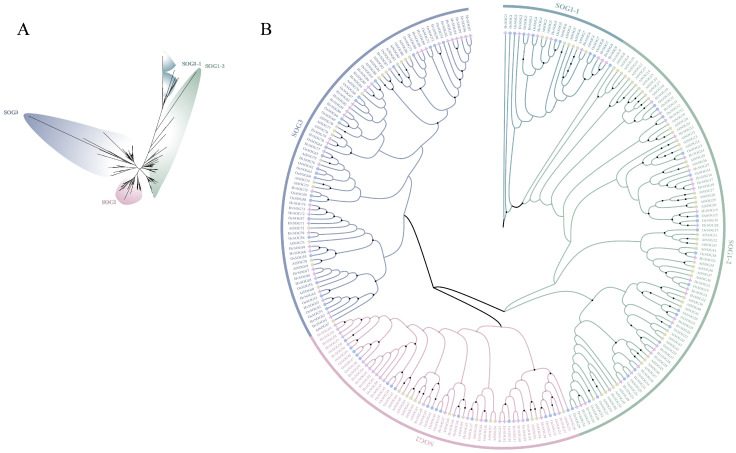
Phylogenetic tree of *SOG* gene families in barley, rice and *arabidopsis*. The different-colored branches and arcs represent different subfamilies. An unrooted phylogenetic tree was generated using IQ-tree with 257 full-length amino acid sequences of SOG, and the bootstrap was set at 5000 replicates. Different-colored nodes indicate SOG proteins from individual species. The phylogenetic tree was displayed in the form of both an unrooted layout in the upper left corner (**A**) and the circular layout on the right (**B**).

To explore the relationships of SOGs between barley and rice, an unrooted phylogenetic tree was constructed using the full-length amino acid sequences of these SOGs (barley and rice; *arabidopsis* as reference). In total, 86 AtSOGs from *arabidopsis*, 97 HvSOGs from barley and 74 OsSOGs from rice were identified and utilized in this study (Figure 1). Here, we divided these SOGs into three subfamilies (SOG1, SOG2 and SOG3) based on phylogenetic tree structure and gene structure, and the first subfamily was further clustered into two groups (SOG1-1; SOG1-2). Specifically, 36/29 members in barley/rice belong to SOG1, 24/21 to SOG2, and 37/24 to SOG3, respectively. All these proteins possessed only one typical NAM domain. Taken together, barley has more SOGs than rice (particularly SOG1 and SOG3), and shows different SOG subfamily distribution from rice.

### 2.2. Gene Structure and Protein Motif Analysis of SOGs in Barley and Rice

**Figure 2:**
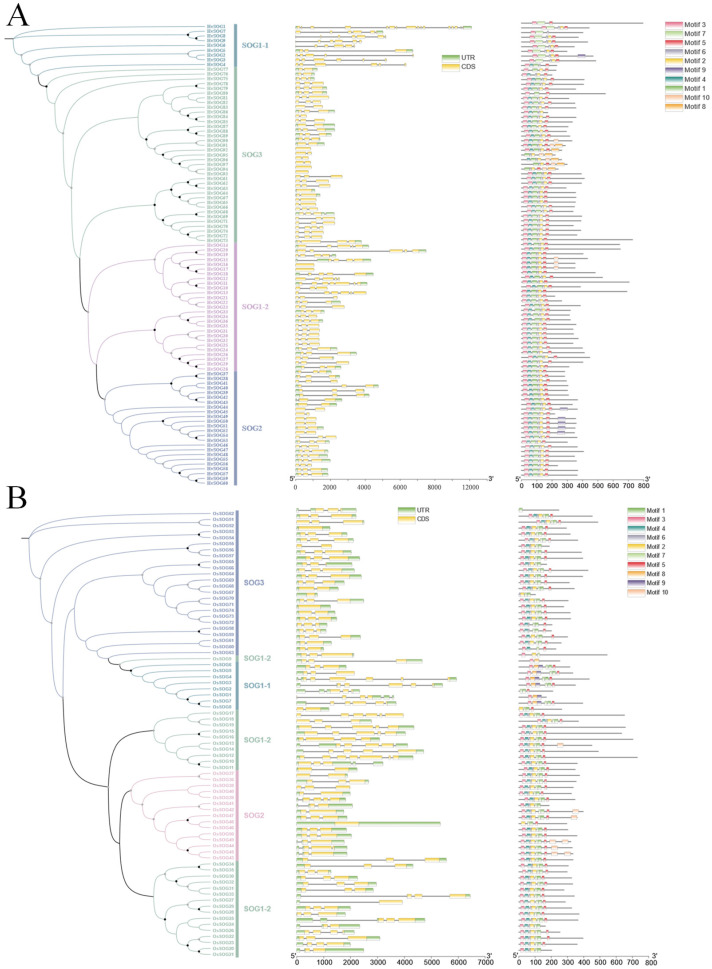
Phylogenetic relationships, gene structures and architecture of conserved protein motifs of *SOGs* from (**A**) barley and (**B**) rice. Left: phylogenetic tree of barley and rice *SOGs*. The tree was generated using IQ-TREE with the bootstrap test replicate set as 5000 times. Center: the exon/intron structure of *SOGs*. Yellow boxes represent exons, black lines represent introns and green boxes represent the UTR regions. Right: the motif composition of barley and rice *SOGs*. Different-colored boxes represent different motifs, with numbers 1–10.

The Gene Structure Display Server (GSDS) was utilized to analyze the gene structures of *HvSOGs* and *OsSOGs* (Figure 2). As shown in Figure 2A, 1–7 exons were found in *HvSOGs*. The *HvSOG1* subfamily genes had 1–7 exons, and 2–3 exons for the *HvSOG2* subfamily genes, and 1–4 exons for the *HvSOG3* subfamily genes. Particularly, the genes in the *HvSOG1* subgroup 1 (*HvSOG1-1*) contained 3–6 exons, while *HvSOG1* subgroup 2 (*HvSOG1-2*) possessed 1–7 exons. Interestingly, rice had similar exon number distribution to barley (Figure 2B).

Furthermore, the schematic structures of all *SOGs* were determined using the results from motif analysis conducted by MEME. The sequence and length information of ten conserved motifs are listed in Supplementary Tables S4 and S5. Usually, the prevailing pattern observed across all these *SOGs* involved motif 3 at the N-terminus and motif 5 at the C-terminus, and there was no consistency among the subfamilies in terms of motif composition, except that the motif composition of the *SOG1* subfamily showed significant differences from other subfamilies, with the order of motif composition being motif 3, 8, 9, 7, 1, 5 (Figure 2). As for the differences between species, most of the *HvSOGs* initiated with motif 3 and ended with motifs 6 and 2 (Figure 2A), while a majority of *OsSOGs* began with motif 3 and ended with motifs 7 and 1 (Figure 2B). We noted that motif 3 (at the N-terminus) was present in all *SOGs* and corresponded to a conserved subdomain essential for DNA binding and dimerization. The C-terminal motifs (e.g., motifs 6 and 2 in barley vs. motifs 7 and 1 in rice) may contribute to differential protein–protein interactions or transactivation activities, potentially explaining species-specific regulatory divergence. In short, the consistent gene structures and conserved motif compositions shared among *SOGs* within the same subfamily provided robust evidence supporting the accuracy of subfamily classifications, and barley exhibited significant differences from rice in motif composition (the end part), which may also be one of the reasons for its greater salt tolerance.

### 2.3. Chromosomal Distribution, Genome Synteny and Gene Duplication of SOGs in Barley and Rice

**Figure 3:**
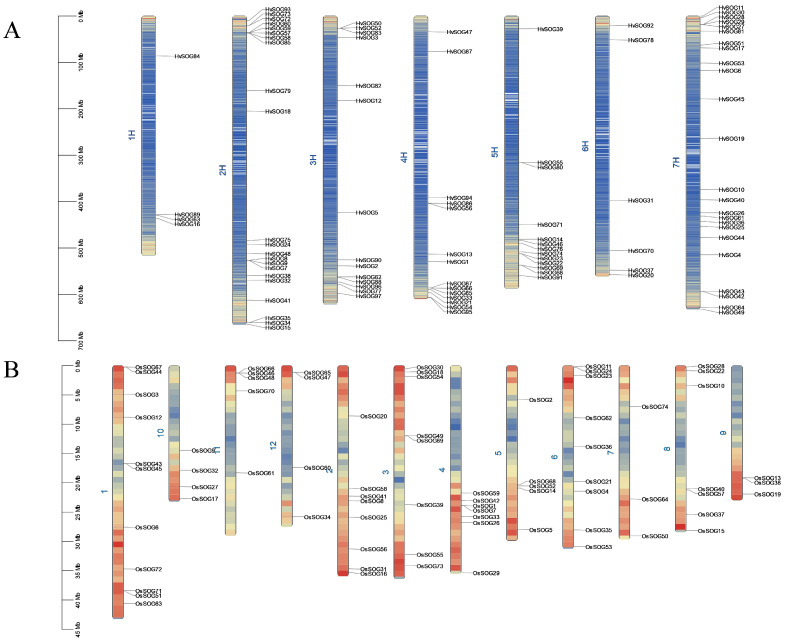
Chromosomal distribution of *SOGs* in barley (**A**) and rice (**B**). Number on left indicates physical location on chromosomes. 1H–7H represent the seven chromosomes of barley, and 1–12 represent the twelve chromosomes of rice.

In total, 97 *HvSOGs* were distributed among seven chromosomes (Figure 3A). Notably, *HvSOGs* tended to cluster, exhibiting a higher distribution density at the ends of each chromosome compared to the central regions (Figure 3A). As for rice, there were 74 *OsSOGs* distributed among 12 chromosomes (Figure 3B). Usually, *OsSOGs* occurred in pairs or triplets, such as *OsSOG43-SOG45* and *OsSOG44-SOG67*, while a significant proportion of genes exhibited random distribution across chromosomes (Figure 3B).

**Figure 4:**
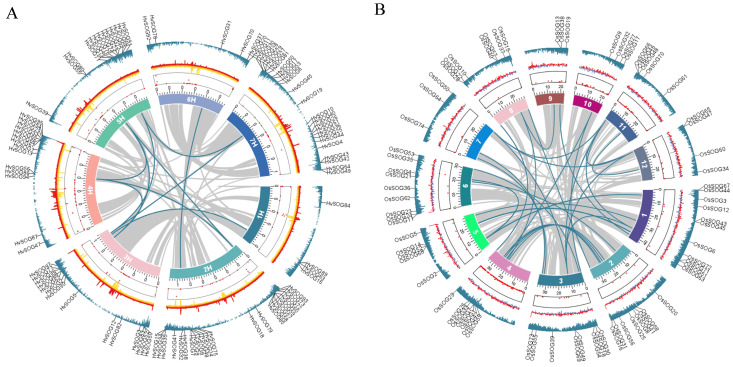
The synteny analysis of *SOG* family in barley (**A**) and rice (**B**). Gray lines indicate all synteny blocks in barley or rice genome, and the blue lines indicate duplicated *SOG* gene pairs. The chromosome number is indicated at the bottom of each chromosome. 1H–7H represent the seven chromosomes of barley, and 1–12 represent the twelve chromosomes of rice.

BLAST (https://www.ncbi.nlm.nih.gov/) and MCScanX (1.0.0) methods were employed to identify duplication events within *SOGs*. In barley, a total of 92 duplication events were detected, including 12 segmental, 21 tandem and 59 dispersed duplication events (Table 1 and Figure 4A). Compared to barley, rice employed a completely distinct method of gene duplication, and only 67 duplication events were identified, with 39/3/25 segmental/tandem/dispersed duplication events (Table 1 and Figure 4B). Additionally, no duplication events were detected within a single chromosome for *SOGs* in either barley or rice; all duplication events occurred between different chromosomes.

**Figure 5:**
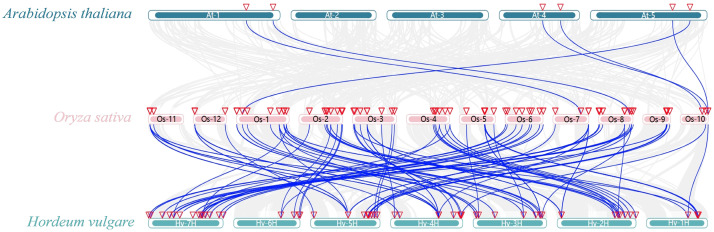
Synteny analysis of *SOGs* between barley, rice and *arabidopsis*. Gray lines in the background indicate the collinear blocks within three species, and the blue lines highlight the syntenic *SOG* gene pairs. At1–At5 represent the five chromosomes of *arabidopsis*; Os1–Os12 represent the twelve chromosomes of rice; Hv1–Hv7 represent the seven chromosomes of barley.

Furthermore, we performed synteny analysis of *SOG* gene pairs among barley, rice and *arabidopsis* genomes. The results showed that six *AtSOGs* and 72 *HvSOGs* exhibited a syntenic relationship with *OsSOGs* (Figure 5), suggesting that these genes might be potentially involved in the evolution of the *SOG* gene family. To assess the selective evolutionary pressure on *SOGs*, we calculated the Ka values, Ks values and Ka/Ks ratio for homologous genes in barley and rice. Consistently, the duplicated *SOG* gene pairs had a Ka/Ks ratio less than 1 in both barley and rice (Table 2 and Tables S6 and S7). For barley, the majority of duplicated *HvSOG* gene pairs had a 0.55 Ka/Ks ratio, and the mean value was 0.63 (Ka/Ks ratio). However, the Ka/Ks value of 0.45 accounted for the highest proportion of *OsSOG* gene pairs, with the mean value being only 0.38 (Ka/Ks ratio) in rice. Clearly, the mean value of *SOG* gene pairs (Ka/Ks ratio) was much lower than *HvSOG* gene pairs. Thus, these results indicated that both *HvSOGs* and *OsSOGs* may have undergone purifying selection pressure during evolution, whereas rice may have suffered stronger purifying selection pressure compared to barley.

### 2.4. Stress-Related Cis-Elements in the Promoters of HvSOGs and OsSOGs

**Figure 6:**
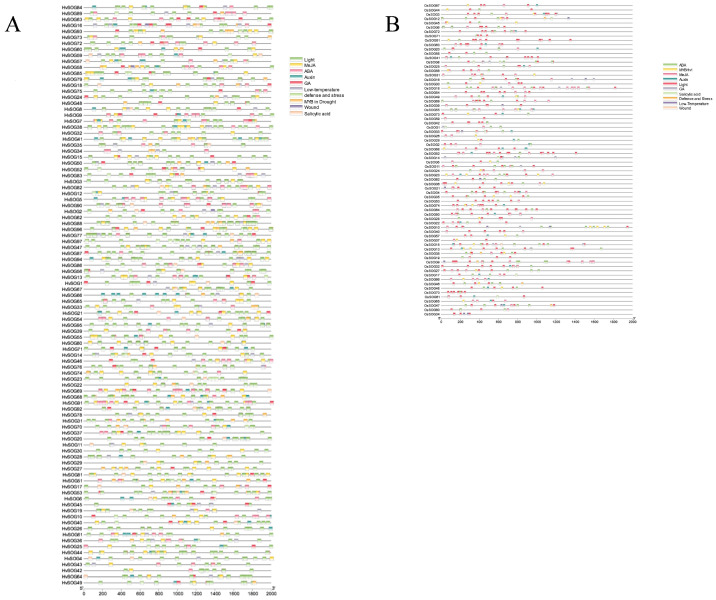
Predicted cis-elements in promoters of barley (**A**) and rice (**B**). Promoter sequences (−2000 bp) of each *SOG* were analyzed by PlantCARE. The scale bar at the bottom (in base pairs, bp) indicates the distance upstream from the translation start site.

To elucidate the potential functions and regulatory mechanisms of *HvSOGs* and *OsSOGs*, cis-elements in the promoter regions (2 kb upstream from ATG) were conducted by using PlantCare. Here, 97 *HvSOGs* and 74 *OsSOGs* were characterized with cis-elements including ABRE, LIRE, GARE, JARE and LTRE, involved in ABA, GA, light, MeJA and low-temperature responses, respectively (Figure 6 and Tables S8 and S9). In barley, 97 *HvSOGs* (100%) exhibited LIRE cis-elements, 92 *HvSOGs* (94.85%) displayed ABRE cis-elements, 89 *HvSOGs* (91.75%) had JARE cis-elements, 60 *HvSOGs* (61.86%) contained GARE cis-elements, and 57 *HvSOGs* (58.76%) carried LTRE cis-elements (Figure 6A and Table S8). As for rice, 72 *OsSOGs* (97.30%) had LIRE cis-elements, 63 *OsSOGs* (85.14%) possessed ABRE cis-elements, 56 *OsSOGs* (75.68%) showed JARE cis-elements, 11 *OsSOGs* (14.86%) carried GARE cis-elements, and 11 *OsSOGs* (14.86%) had LTRE cis-elements (Figure 6B and Table S9). Therefore, the cis-element results showed that most *SOGs* in both barley and rice can respond to various environmental stresses, and the proportion of *SOGs* with stress-related cis-elements in barley was significantly higher than that in rice.

### 2.5. Expression Profiles of HvSOGs and OsSOGs in Different Tissues Under Salt Stress

**Figure 7:**
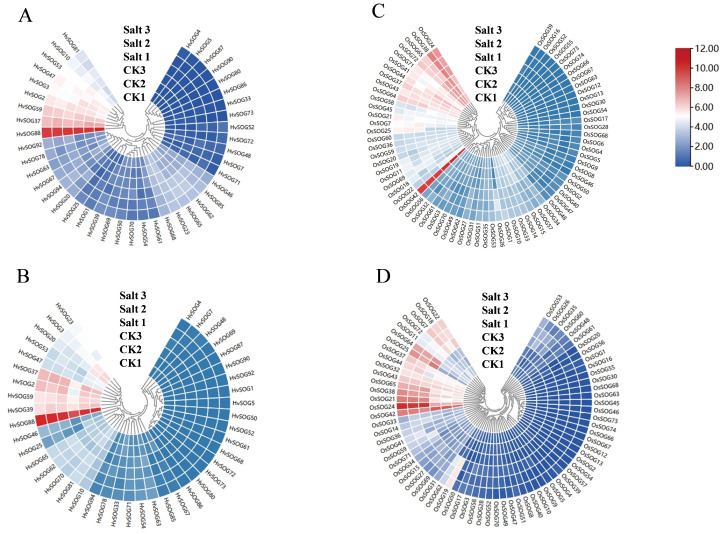
Expression pattern of *SOGs* in control and salt conditions. (**A**) Expression profiles of the *HvSOGs* in root and shoot (**B**) tissues. (**C**) Expression profiles of the *OsSOGs* in root and shoot (**D**) tissues. CK1-3 means three replicates under control condition. T1-3 means three replicates under salt condition. The color scale represents relative expression levels from high (red color) to low (blue color).

The expression profiles of *HvSOGs* and *OsSOGs* were investigated in shoot and root tissues of barley and rice under 100 mM salt stress for 9 d (Figure 7 and Figure S1A and Tables S10 and S11). In barley, salt treatment did not cause a significant gene expression change in most *HvSOGs*, with only 2/4 genes being up-or down-regulated in shoot and 3/1 genes being up-/down-regulated in root. Among these differentially expressed *HvSOGs*, *HvSOG20* was especially up-regulated (3.72-fold) in the roots but down-regulated (0.45-fold) in the shoots (Figure 7A,B), which may play a certain role in the salt tolerance of barley. In contrast, a large number of *OsSOGs* in rice were up- or down-regulated in response to salt stress (Figure 7C,D). In the roots of rice, 33.87% of *OsSOGs* were differentially expressed, and most of these DEGs were down-regulated. However, 51.67% of *OsSOGs* were differentially expressed in shoot tissues, with most of these DEGs being up-regulated. Taken together, these results are consistent with the known higher salt tolerance of barley compared to rice, as barley exhibited fewer differentially expressed *SOGs* under salt stress. However, further functional studies are needed to establish a direct causal relationship.

### 2.6. Homologous Gene Similarity of SOGs in Evolution Compared with Salt-Tolerant Plants

To better understand the possible reasons for functional differences in salt tolerance between *HvSOGs* and *OsSOGs*, we conducted a global alignment BLAST among barley, rice and sea barleygrass (*Hordeum marinum*; a representative extremely salt-tolerant plant), and calculated the average similarity of *HvSOGs* and *OsSOGs* compared with sea barleygrass. Based on the 97 *HvSOGs* and 74 *OsSOGs,* 158 *HmSOGs* were identified in sea barleygrass. Interestingly, barley *HvSOGs* showed high sequence consistency with sea barleygrass, with an average similarity 63% (Figure S1B). In contrast, rice *OsSOGs* exhibited only 39% similarity with sea barleygrass (Figure S1C), which was significantly lower than barley. Overall, *SOGs* were relatively conserved in salt-tolerant species, and the loss and functional divergence of gene members during the evolutionary process of rice could be one of the reasons for salt sensitivity.

### 2.7. The Up-Regulated Gene OsSOG17 Positively Regulates Salt Tolerance in Rice

**Figure 8:**
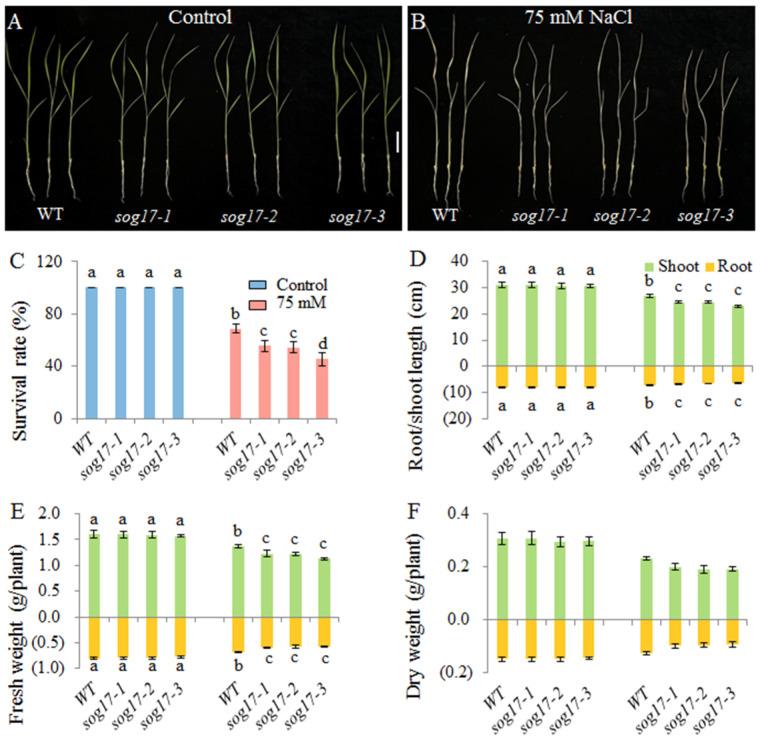
Growth performance of *sog17* mutant lines and WT seedlings under control and salt treatment. (**A**) Pictures of rice seedlings after 7 days of control and (**B**) salt treatment. (**C**) Survival rates of rice seedlings under control and salt treatment after 7 days (%). (**D**) Root and shoot lengths measured under control and salt treatment. (**E**) Fresh and (**F**) dry weights of roots and shoots measured under control and salt treatments. Different letters indicate significant differences at *p* < 0.05 based on one-way ANOVA (*n* = 6). Scale bars, 5 cm.

To verify the reliability of the genes identified through combined analysis of gene family identification and gene expression profiling, we performed functional validation of *OsSOG17*, a differentially expressed gene (up-regulated) under salt stress (Table S11). Using CRISPR-Cas9, we obtained loss-of-function mutants of *OsSOG17*. Hydroponic experiments were conducted using three mutant types and the wild type. Under normal growth conditions, no significant differences in growth were observed between the mutants and the wild type (WT) (Figure 8A). Following 7 days of salt treatment, plant growth was consistently more inhibited in all mutant lines than in the WT under the 75 mM NaCl condition (Figure 8B). Compared with the WT, the *sog17* mutants exhibited significantly lower survival rates, as well as shorter root and shoot lengths, under salt stress (Figure 8C,D). In addition, the mutants accumulated less biomass than the WT under salt stress, as indicated by both fresh and dry weights (Figure 8E,F).

**Figure 9:**
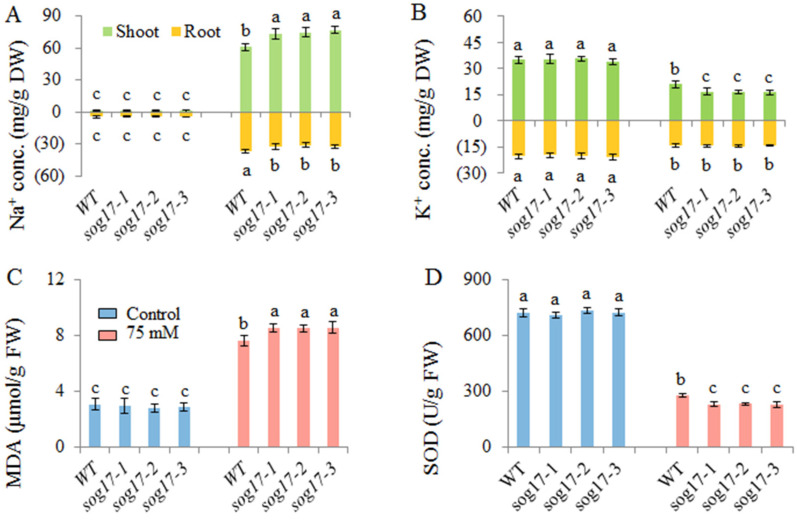
Ion concentration and enzyme activity of *sog17* mutant lines and WT seedlings under control and salt treatment. (**A**) Na^+^ and (**B**) K^+^ concentrations of mutant lines and WT seedlings under control and salt treatments. (**C**) Accumulation of malondialdehyde (MDA) and (**D**) the SOD activities in mutant lines and WT seedlings under control and salt treatments. Different letters indicate significant differences at *p* < 0.05 based on one-way ANOVA (*n* = 6).

To assess the impact of *OsSOG17* on tissue ion concentrations, we measured Na^+^ and K^+^ levels using inductively coupled plasma optical emission spectroscopy (ICP-OES). Under normal conditions, no significant differences in tissue Na^+^ and K^+^ concentrations were observed between the mutants and WT plants (Figure 9A,B). After 7 days of salt treatment, root Na^+^ concentrations were significantly higher in the WT than in the three mutant lines under 75 mM NaCl (Figure 9A). In contrast, shoot Na^+^ concentrations were lower in the WT than in the mutants under salt treatment (Figure 9A). Under salt stress, K^+^ concentrations were significantly reduced in plant tissues. In the roots, no significant differences were observed between the WT and the mutants. In contrast, K^+^ concentrations in the shoots of the mutants were significantly lower than those in the WT under salt stress (Figure 9B). We further measured the malondialdehyde (MDA) and superoxide dismutase (SOD) contents in the mutants and WT under salt stress. Under control conditions, no significant differences in MDA or SOD contents were observed between the WT and the mutants (Figure 9C,D). Salt stress significantly increased MDA content in the plants; however, the increase was significantly greater in the *sog17* mutants than in the WT (Figure 9C). Salt stress also significantly decreased SOD activity, and the reduction was significantly more pronounced in the *sog17* mutants than in the WT (Figure 9D). These results indicate that knockout of *sog17* significantly reduces salt tolerance in rice.

## 3. Discussion

Both barley and rice belong to the Poaceae family and are diploid crops, and they share a similar number of many homologous conserved gene families, such as the *HKT*, *NHX*, and *SOS* gene families. These families are central to plant salt tolerance: *HKTs* mediate Na^+^ transport and recirculation, *NHXs* compartmentalize Na^+^ into vacuoles, and *SOSs* coordinate Ca^2+^-dependent Na^+^ exclusion. The fact is that barley and rice possess comparable copy numbers of these core ion-homeostasis gene families. Our study reveals that the *SOG* gene family, a plant-specific transcription factor family primarily known for DNA damage responses, shows striking divergence between barley and rice in terms of its gene number (97 vs. 74), and subfamily distribution (particularly *SOG1* and *SOG3*). These features suggest that the *SOG* family may act as a species-specific transcriptional regulator that fine-tunes the expression of downstream salt-tolerance genes, including potentially *HKT*, *NHX*, or *SOS* genes. This could enable barley to achieve more efficient regulation of ion homeostasis under stress with lower energy expenditure. In contrast, rice, despite having a similar core ion transporter repertoire, possesses fewer *SOGs*. This may result in a less flexible transcriptional response. Thus, while the *HKT*, *NHX*, and *SOS* families provide a conserved basal framework for ion homeostasis, the *SOG* family appears to have undergone functional divergence that may contribute to the superior salt tolerance of barley. Interestingly, the result of homologous gene similarity in *HvSOGs* and *OsSOGs* compared with ancient salt-tolerant plants also confirmed the above assumption, with the loss of *SOG* gene number in rice during the evolutionary process causing the salt sensitivity of rice (Figure S1), indicating the importance of plant *SOGs* in the salt stress adaptation.

The differences in gene and protein structures among *SOG* subfamilies may also contribute to functional divergence. In general, intron-rich genes are typical of eukaryotes, whereas intronless genes are more common in prokaryotes [31]. However, in our study, we found that 6.2% of *HvSOGs* and 5.4% of *OsSOGs* lack introns (Tables S2 and S3). Although these proportions are modest, they are noteworthy because intronless genes often experience stronger functional constraints during evolution. Indeed, it has been reported that in *arabidopsis*, certain intronless genes belonging to families such as AP2, EF-hand, bZIP, FAD-binding, and C2 are more likely to be involved in salt stress responses [32]. The absence of introns can have several mechanistic consequences. First, intronless genes generally undergo a simpler transcriptional process, as they do not require splicing. This may enable faster transcription and more rapid mRNA accumulation, which could be advantageous under sudden stress conditions where timely gene expression is critical. Second, the lack of introns often reduces the potential for alternative splicing, thereby limiting regulatory complexity but possibly ensuring a more predictable and consistent protein product. Third, intronless genes frequently undergo stronger purifying selection, as the absence of introns removes a common source of neutral variation, making any non-synonymous mutation more likely to be subject to direct selection. Our observation that a subset of *SOGs* are intronless raises the possibility that these genes have been evolutionarily streamlined for rapid and energy-efficient responses. This idea aligns with our proposed “shoot energy-saving strategy” for salt tolerance in barley: minimizing unnecessary transcriptional complexity may conserve energy and allow faster activation of stress-defense mechanisms. Interestingly, we also observed that most of these intronless *SOGs* in both species contain multiple stress-related cis-elements in their promoters (e.g., ABRE, LIRE, JARE), further supporting their potential role in immediate stress perception and response.

Barley exhibited significant differences from rice in motif composition, particularly at the C-terminus. This may be one of the reasons for its stronger salt tolerance. In NAC transcription factors, transactivation activity is primarily dependent on the C-terminal region, which contains group-specific motifs and exhibits a high degree of intrinsic disorder. Domain-swapping experiments have shown that replacing the C-terminal transactivation domain of ANAC019 with other NAC C-termini abolishes its ABA signaling function, whereas swapping the conserved N-terminal DNA-binding domain retains its biological function [33]. This indicates that functional divergence is largely determined by C-terminal variation. These observations from *arabidopsis* provide a strong framework for interpreting our study: the divergent C-terminal motifs between barley and rice SOGs may directly influence transactivation capacity, downstream target recognition, and ultimately salt stress responses.

In plant genomes, tandem and segmental duplication were two important mechanisms for gene amplification. The prevalence of tandem duplication demonstrated an effective adaptation strategy employed by plants to withstand varying environments during the evolutionary process [34]. In our study, we found *HvSOGs* had more tandem duplication events compared to *OsSOGs* (Table 1), which may lead to barley’s stronger salt tolerance than rice. Interestingly, most of the *HvSOGs* with tandem duplication events were differentially expressed genes, which allowed rapid gene activation and timely response to diverse salt stress. On the other hand, *OsSOGs* possessed much more segmental duplication events compared to *HvSOGs* (Table 1). Usually, segmental duplication typically entailed larger blocks of DNA sequences and was more prevalent in stable genomes. The rice genome, being smaller and more compact, may exhibit higher genomic stability, and this may be the reason for the predominant status of segmental duplication events in the expansion of *OsSOGs.* Additionally, the distribution of *SOGs* on chromosomes also showed a significant difference between barley and rice, with *HvSOGs* tending to cluster and *OsSOGs* appearing in couples or triples, which underscored the divergent modes of expansion in these two species. This variance likely contributed to the emergence of multiple clusters of *SOGs* with analogous sequences and functions in barley.

A previous study indicated that functional elements, including genes, were primarily subject to substantial selective constraints [35]. Typically, the process of purifying selection had a strong impact on genetic diversity at both directly affected and neutral-linked sites, and this process is essential for shaping genomic diversity in natural populations. In our study, the calculated Ka/Ks values of 0.63 for barley and 0.38 for rice indicated that both *HvSOG* and *OsSOG* gene families had undergone purifying selection during evolution (Tables S6 and S7). This also implied that during the evolution of land plants from seawater to land, due to the improvement in their living environment, some *SOGs* began to be lost or become functionally divergent. Meanwhile, rice had a lower rate of non-synonymous substitutions compared to barley, and may suffer stronger natural purifying selection pressure. However, strong purifying selection could limit adaptive evolution. While purifying selection maintained stability, it may also restrict the acquisition of new beneficial mutations that could enhance salt tolerance. In contrast, a slightly relaxed purifying selection (as seen in barley) allowed more non-synonymous substitutions, some of which may confer advantages under stressful environments such as high salinity. This was consistent with the evolutionary history of barley, which originated in harsh environments (e.g., the Tibetan Plateau) and was frequently exposed to abiotic stresses. Thus, the degree of salt stress that rice may experience during its life cycle was relatively low, unlike barley which was prone to salt stress in arid environments.

Furthermore, following the analytical framework established by Ninkuu et al. (2023) [36] for the benzoxazinoid gene family in rice, we extended our analysis to interpret the functional relevance of chromosomal distribution and motif conservation. In our study, the observed clustering of *HvSOGs* toward the distal ends of barley chromosomes may facilitate rapid transcriptional activation under salt stress, analogous to the functionally associated chromosomal colocalization reported for the benzoxazinoids genes. Similarly, the distinct motif architectures between barley and rice *SOGs*, particularly the C-terminal differences, likely reflect functional divergence between the two species, supporting the notion that conserved structural features can provide functional clues for stress adaptation. The universal presence of motif 3 at the N-terminus across all *SOG* reinforces its essential role in maintaining the integrity of the NAM domain.

Accumulating evidence has demonstrated that cis-elements in promoter regions play a crucial role in regulating gene expression during plant growth and in response to environmental stimuli. Our analysis revealed that both *HvSOGs* and *OsSOGs* harbor various types of stress-related cis-elements, including LIRE, ABRE and JARE. Most gene promoters contained at least one such element, indicating that these *SOGs* are not only involved in salt tolerance but may also play broader roles in multiple abiotic and biotic stress responses (Figure 6). The presence of ABRE elements is particularly noteworthy, as they are known to mediate abscisic acid signaling, a central pathway in osmotic and salt stress adaptation. Similarly, JARE elements suggest possible crosstalk with jasmonate signaling, which is often associated with defense against biotic stress but has also been implicated in salt tolerance. The coexistence of multiple stress-related cis-elements within the same promoter implies that individual *SOGs* might be integrated into complex regulatory networks, allowing them to respond to diverse environmental cues in a coordinated manner. Interestingly, the proportion of *SOGs* carrying stress-related cis-elements was significantly higher in barley than in rice. This disparity suggests that *HvSOGs* are under stronger transcriptional regulation by stress signals, potentially enabling a more nuanced and rapid response to adverse conditions. The higher cis-element richness in barley may also contribute to the greater complexity of its regulatory pathways under salt stress, which could partly explain why barley exhibits stronger salt tolerance than rice. Our cis-element analysis also has direct implications for the “shoot energy-saving strategy” proposed in this study. The fact that *HvSOGs* possess more stress-related cis-elements does not necessarily mean they are more transcriptionally active under salt stress; rather, they may be more finely tuned. Indeed, our expression data showed that most *HvSOGs* maintain stable expression under salinity, whereas *OsSOGs* undergo massive up-regulation. This suggests that in barley, the presence of cis-elements might allow for precise, context-dependent regulation without constitutive activation, an energy-efficient design.

It was well documented that the gene expression pattern was the most intuitive reflection of the changes in gene response to various signals and provided key evidence to map out gene functionality. Certainly, energy conservation under stress is a well-recognized strategy for maintaining growth and ion homeostasis, whether in comparisons between barley and sea barleygrass (a more salt-tolerant species), among different genotypes of sea barleygrass, or among different rice genotypes. By analyzing the expression patterns of *HvSOGs* and *OsSOGs* in shoots and roots under salt stress in our transcriptome data, we found that the higher salt tolerance of barley relative to rice was mainly attributed to less energy consumption, which was consistent with our previous study [37]. In detail, 33.87% and 51.67% of *SOGs* in rice were differentially expressed in roots and shoots, but not in barley (Figure 7). Particularly, among these differentially expressed *OsSOGs* in shoots, 77.42% of *OsSOGs* were up-regulated and the average change was 9.7-fold, which may consume a lot of energy to fight against salt stress. Interestingly, *AtSOG1* could interact with the transcription factor SnRK1, and its expression was significantly increased in low-energy environments to regulate plant adaptation, while SnRK1 played an important role in abiotic stress. Here, as the homologous gene of *AtSOG1, OsSOG7* was markedly up-regulated (6.07-fold) in shoots to fight against salt stress [26], while the homologous gene in shoots of barley kept a stable expression level under salt stress. Thus, it may be assumed that shoot energy-saving could be a major mechanism for barley to adapt to salt stress.

Integrating bioinformatics analysis with molecular functional validation provides a more compelling approach for identifying key genes involved in stress responses. In this study, we focused on *OsSOG17*, a gene that was significantly up-regulated under salt stress based on transcriptomic data. Through functional characterization, we demonstrated that knockout of *OsSOG17* led to a marked reduction in salt tolerance in rice, indicating that *OsSOG17* acts as a positive regulator of salt tolerance. Notably, *OsSOG17* is predicted to function as an upstream regulator, potentially modulating the expression of downstream genes involved in ion transport and antioxidant defense. For instance, the altered Na^+^ and K^+^ accumulation patterns and the differential changes in MDA and SOD contents in the *sog17* mutants compared with the wild type suggest that *OsSOG17* may govern a broader regulatory network that integrates ion homeostasis and oxidative stress responses (Figure 9). Further investigation into the downstream targets and interacting partners of *OsSOG17* will help elucidate the molecular mechanisms underlying its role in enhancing salt tolerance in rice.

Above all, this research offered an in-depth understanding of the *SOG* gene family in both barley and rice. Meanwhile, we provided a comprehensive comparison of *SOG* family genes in salt tolerance between barley and rice. This was of great significance in further understanding the biological functions of the *SOGs* in crops and their roles in breeding. Of course, deeper gene functional validation needs to be done in future to reveal the exact roles of *SOGs*.

## 4. Materials and Methods

### 4.1. Identification of SOG Family Genes in the Barley, Rice and Arabidopsis Genome

The reference sequences of the *SOG* gene family in *arabidopsis* were downloaded from UniProt (https://www.uniprot.org; 1 March 2024). Genomic sequences and annotation data for barley, rice and *arabidopsis* were obtained from the Ensembl Plant database (https://plants.ensembl.org/index.html; 1 March 2024). The homologous genes of *AtSOGs* in barley and rice genome were blasted in the reference genome of cv. Morex and Nipponbare, respectively. Initial identification of HvSOG, OsSOG and AtSOG was conducted through the Hidden Markov Model (HMM) and BLASTP program. NAM structural domain model files were retrieved from the Pfam database [38] (http://pfam.janelia.org/; 1 March 2024) for local BLASTP searches (1 × 10^−5^). All candidate sequences of *SOGs* were validated using the SMART database [39] (http://smart.embl-heidelberg.de/; 1 March 2024) and the NCBI Conserved Domain database [40]. Subfamily members were named based on their arrangement order on chromosomes of each genome. In addition, the number of amino acids, molecular weight (MW) and isoelectric point (pI) of SOGs were calculated by the ExPASy tool (https://www.expasy.org/; 1 March 2024).

### 4.2. Phylogenetic Analysis and Classification of SOG Gene Family in Barley and Rice

Multiple sequence comparisons of 97 *HvSOGs*, 74 *OsSOGs* and 86 *AtSOGs* amino acid sequences were performed using the ClustalW tool (version 1.2.4) with default parameters. According to the results of the comparison, phylogenetic trees were constructed using IQ-tree [41] by the Maximal Likelihood (ML) method, with the parameters of the default model and 5000 bootstrap replicates. The same method was also performed to construct the unrooted ML trees for all SOG protein sequences from barley and rice.

### 4.3. Identification of Motif Compositions and Gene Structures

The Multiple Expectation Maximization for Motif Elicitation (MEME, https://meme-suite.org/meme/; 1 March 2024) online tool was utilized to identify the conserved motifs of SOG proteins. Parameters included an arbitrary number of repetitions, a maximum of 10 motifs, and an optimal motif length ranging from 6 to 100 amino acid residues. The exon–intron structures of *HvSOG* and *OsSOG* gene families were analyzed by the Gene Structure Display Server online tool (GSDS: http://gsds.cbi.pku.edu.cn; 1 March 2024).

### 4.4. Chromosomal Location and Gene Duplication Analyses

The chromosomal locations of all *HvSOGs* and *OsSOGs* were mapped to 7 and 12 chromosomes using TBtools-II (Toolbox for Biologists) based on the physical location information from their genome databases. Gene synteny analysis was performed in barley and rice as well as among the three species using the MCScanX (1.0.0) with default parameters to categorize the replication modes of the *SOGs*, including segmental, tandem and transposon duplications. The homology relationships among the three species were visualized using Tbtools (version 1), and the non-synonymous (Ka) and synonymous (Ks) substitution values of each pair of homologous *HvSOGs* and *OsSOGs* were calculated using KsKs_Calculator 2.0.

### 4.5. Cis-Elements in Promoter Regions of HvSOGs and OsSOGs

The upstream sequences (2000 bp) preceding the initiation codon (ATG) of each *SOG* were retrieved from the genome sequences of barley and rice. These sequences were subsequently analyzed to identify the types, positions and distributions of cis-elements in the promoter regions using PlantCARE (http://bioinformatics.psb.ugent.be/webtools/plantcare/html/; 1 March 2024). The results were then visualized using Tbtools (version 1).

### 4.6. Expression Patterns of HvSOGs and OsSOGs

Transcriptome data of barley and rice treated with 100 mM NaCl were acquired from the previous research by Fu et al. [26], which was accessible in the NCBI repository under project ID PRJNA546269. Differential expression analyses of *HvSOGs* and *OsSOGs* were conducted utilizing the DESseq2 R package and heatmaps were generated using TBtools (version 1). The thresholds for defining differentially expressed genes (DEGs): ∣log2(Fold Change)∣ > 1 and False Discovery Rate (FDR) < 0.001.

### 4.7. Homologous Gene Similarity Analyses of SOGs in Evolution

The homologous genes of *SOGs* in sea barleygrass were identified. The genome data and annotation for sea barleygrass were acquired from the CNCB (National Genomics Data Center) (https://ngdc.cncb.ac.cn; 12 March 2024), originating from a previous study by Liu et al. [42]. After BLASTP (https://www.ncbi.nlm.nih.gov/) analysis of the sea barleygrass genome, interspecific gene synteny analysis was performed among barley, rice, and sea barleygrass. Global alignment and evolutionary analysis were utilized to compare the sequences of homologous genes of *SOGs*.

### 4.8. Generation and Phenotypic Analysis of Sog17 Mutants

To generate loss-of-function mutants of *OsSOG17*, CRISPR/Cas9 gene editing technology was employed. Three independent mutant lines were selected for subsequent analyses. All plants were grown in a growth chamber under controlled conditions. For phenotypic evaluation under salt stress, 10-day-old seedlings of the wild type (WT) and mutant lines were transferred to hydroponic culture containing 1/2 Kimura B nutrient solution for 20 days, and then treated with 0 (control) or 75 mM NaCl for 7 days [43]. Plant growth was documented photographically, and survival rates were recorded. Root and shoot lengths were measured, and fresh weights were determined immediately after harvest; dry weights were obtained after oven-drying at 80 °C for 48 h. For ion content analysis, Na^+^ and K^+^ concentrations in roots and shoots were measured using inductively coupled plasma optical emission spectroscopy (ICP-OES). Lipid peroxidation was assessed by measuring malondialdehyde (MDA) content, and superoxide dismutase (SOD) activity was determined using commercial assay kits (Nanjing Jiancheng, Nanjing, China). All experiments were performed with six biological replicates.

## 5. Conclusions

In this study, genome-wide identification and characterization of *SOGs* were performed in barley and rice. In total, 97 *HvSOGs* and 74 *OsSOGs* were identified from each genome, which could be classified into three subfamilies. Phylogenetic analysis and evolutionary analysis of homologous *SOGs* provided valuable insights into the evolutionary history of this gene family. Additionally, genome synteny, cis-elements, gene structure, duplication events, and expression patterns were determined, and a genome-wide comparison of *SOG* family genes in relation to salt tolerance was conducted between barley and rice. Notably, a differentially expressed *SOG* under salt stress in rice was selected for functional validation, which confirmed its positive regulatory role in salt tolerance. Collectively, these findings provide important clues for further understanding the biological functions of *SOGs* in barley and rice, particularly with respect to salt tolerance.

## Figures and Tables

**Table 1:** The duplication types of *HvSOGs* and *OsSOGs*.

Gene	Segmental Duplication	Tandem Duplication	Dispersed Duplication
*HvSOGs*	12	21	59
*OsSOGs*	39	3	25

**Table 2:** The frequency of Ka/Ks values for *HvSOGs* and *OsSOGs*.

Gene	Range				
	0–0.2	0.2–0.4	0.4–0.6	0.6–0.8	0.8–1.0
*HvSOGs*	1	0	3	2	2
*OsSOGs*	3	7	11	0	1

## Data Availability

All data analyzed in this study were included in the manuscript and its Supplementary Materials. RNA-Seq data were downloaded from the NCBI database (BioProject accession no. PRJNA546269).

## Supplementary Materials

The following supporting information can be downloaded at: https://www.mdpi.com/article/10.3390/plants15111620/s1, Table S1: The information of 86 *AtSOGs* identified in this study; Table S2: The information of 97 *HvSOGs* identified in this study; Table S3: The information of 74 *OsSOGs* identified in this study; Table S4: Conserved amino acid motifs of HvSOGs; Table S5: Conserved amino acid motifs of OsSOGs; Table S6: Ka/Ks values of *HvSOG* genes; Table S7: Ka/Ks values of *OsSOG* genes; Table S8: Cis-elements of promoter for 97 *HvSOGs*; Table S9: Cis-elements of promoter for 74 *OsSOGs*; Table S10: The expression profiles of the *HvSOGs* under control and salt conditions; Table S11: The expression profiles of the OsSOGs under control and salt conditions; Figure S1: The expression level of *SOGs* and sequence similarity of *SOG* homologous gene pairs among barley, rice and sea barleygrass.
