# Supplementary material for: Divergent Roles of SOG Family Genes in Salt Tolerance: A Comparative Genomics Study Between Barley and Rice
Source: Plants (Basel). 2026 May 25;15(11):1620. doi: 10.3390/plants15111620 (PMC13258844; doi:10.3390/plants15111620)
Supplement: Supplementary file 1 [file plants-15-01620-s001.zip › Supplementary Figure.pptx]

## Slide 1
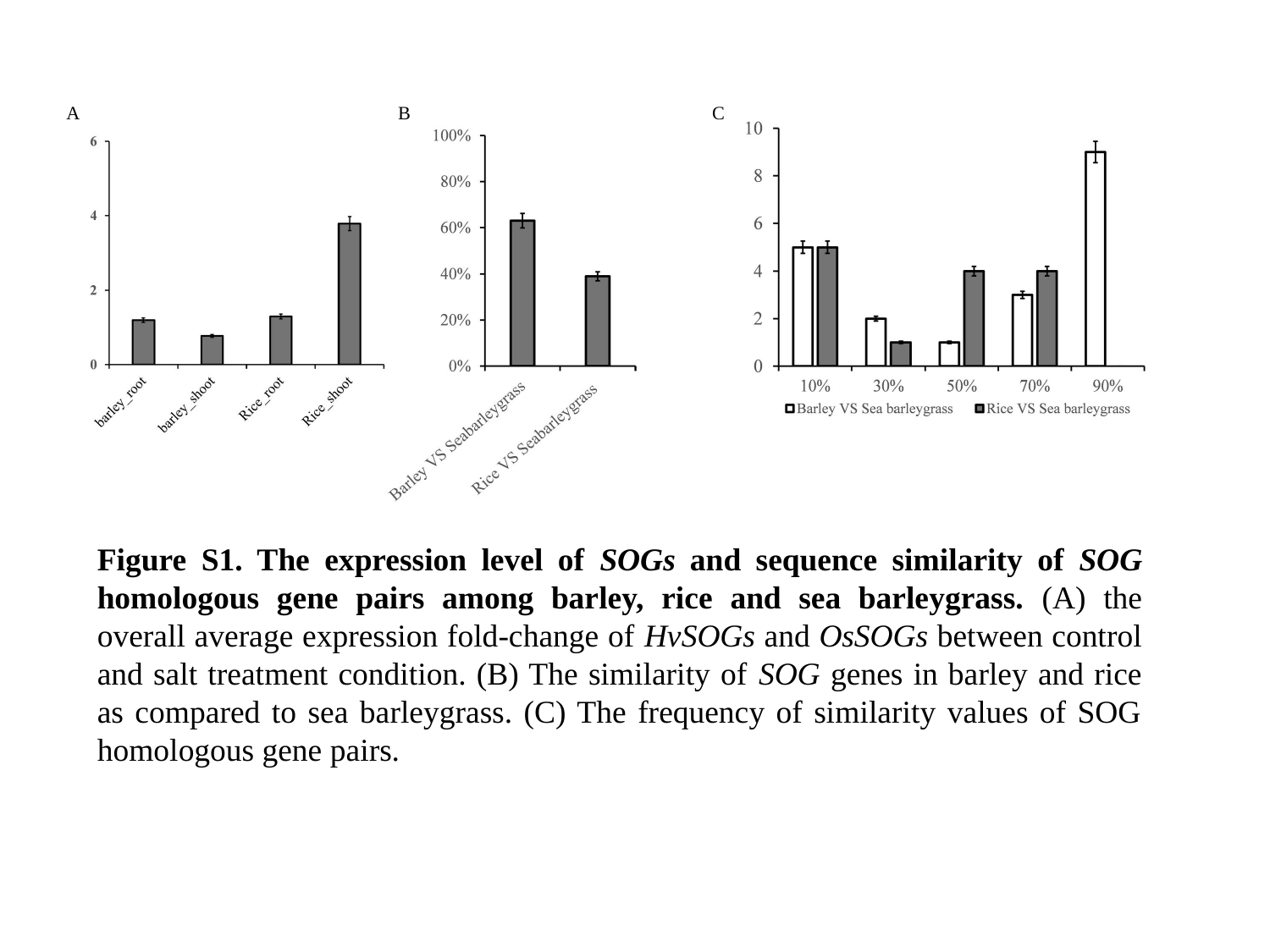

Figure S1. The expression level of SOGs and sequence similarity of SOG homologous gene pairs among barley, rice and sea barleygrass. (A) the overall average expression fold-change of HvSOGs and OsSOGs between control and salt treatment condition. (B) The similarity of SOG genes in barley and rice as compared to sea barleygrass. (C) The frequency of similarity values of SOG homologous gene pairs.
